# Electric bicycle versus conventional bicycle crashes: comparative analysis of injury patterns, severity, and the modifying effect of body mass index

**DOI:** 10.1007/s00068-026-03254-w

**Published:** 2026-08-14

**Authors:** D. Bauer, J. P. Maier, L. Holzfurtner, C. Pietsch, F. C. Wagner, H. Schmal, B. Erdle, N. Mühlenfeld

**Affiliations:** 1 Department of Orthopedics and Trauma Surgery, Faculty of Medicine, Medical Centre - Albert-Ludwig’s- University of Freiburg, Albert-Ludwig’s-University of Freiburg, Freiburg, Germany; 2 Department of Orthopedic Surgery, University Hospital Odense, Sdr. Boulevard 29, Odense C, 5000 Denmark

**Keywords:** Electric bicycle, Body mass index, Cycling injuries, Injury patterns

## Abstract

**Purpose:**

E-bike trauma is often presumed to result in greater injury severity than conventional cycling, yet evidence remains inconsistent. Furthermore, the impact of factors such as Body Mass Index (BMI) on injury patterns is poorly understood. This study therefore evaluated whether e-bike trauma is associated with increased overall severity or distinct regional injury patterns.

**Methods:**

This retrospective single-center cohort study analyzed 261 adult trauma-bay patients (e-bike *n* = 37; conventional bicycle *n* = 224). Primary endpoints included Injury Severity Score (ISS) and regional maximum Abbreviated Injury Scale (AIS) scores. Secondary endpoints included physiological burden (SAPS II) and resource utilization (LOS, TISS-10). Multivariate logistic regression was used to identify independent predictors of intracranial hemorrhage, e-bike riding and extremity fractures. Event counts and events-per-variable (EPV) ratios are reported for each model.

**Results:**

E-bike riders had a significantly higher BMI than conventional cyclists (26.1 vs. 24.6 kg/m²; *p* = 0.029). However, the absolute difference of approximately 1.5 kg/m² is modest, and both group medians fall within the normal-to-overweight range. No statistically significant differences were observed in ISS, physiological burden, or resource utilization. Conventional cyclists had a higher proportion of patients with AIS Pelvis/Extremities ≥ 3 (14.3% vs. 5.4%; *p* = 0.045). In the multivariate binary logistic regression models, ISS (OR 1.042; 95% CI 1.014–1.071; *p* = 0.003) and BMI (OR 0.901; 95% CI 0.840–0.967; *p* = 0.004) were independent predictors of extremity fractures; age was not (OR 1.012; *p* = 0.134). Age was the only independent predictor of intracranial hemorrhage (ICH) (OR 1.033; 95% CI 1.017–1.050; *p* < 0.001). BMI was the only independent predictor of e-bike riding (OR 1.130; 95% CI 1.037–1.230; *p* = 0.005).

**Conclusion:**

In this selected cohort of trauma-bay patients, e-bike-related injuries showed no statistically significant difference in overall injury severity or treatment burden compared to conventional cycling injuries. Rather than being inherent to the bicycle type, injury patterns appear driven by overall trauma severity (ISS) and patient age, while higher BMI independently predicts both e-bike riding (demographic self-selection) and a reduced risk of extremity fractures. Larger prospective studies are required however, before conclusions regarding comparative e-bike injury risk can be further drawn.

## Introduction

Despite continuous improvements in trauma systems, registry-based quality assurance, and structured emergency algorithms, acute trauma remains a major clinical and socioeconomic challenge worldwide [1–3]. Early risk stratification using validated trauma scoring systems such as the Injury Severity Score (ISS), trauma indices, and shock indices is fundamental for outcome prediction and allocation of resources in severely injured patients [4]. Accurate early assessment is particularly relevant in mechanisms with heterogeneous injury distributions, including cycling-related trauma.

Cycling-related injuries represent a substantial proportion of trauma admissions [5], and the rapid expansion of electrically assisted bicycles (e-bikes) has substantially altered the epidemiology of bicycle-associated injuries. Recent comparative analyses demonstrate that e-bike riders differ demographically from conventional cyclists and may exhibit distinct injury distributions [6–8]. Spörri et al. reported differences in injury patterns between electric bicycles, conventional bicycles, and motorcycles, highlighting variations in affected body regions and age distribution [9]. Similarly, Berk et al. observed increased hospitalization rates among e-bike users, although without consistently higher global injury severity scores [10]. Database analyses further suggest that injury mechanisms and severity metrics in e-bike crashes differ from those of other two-wheeler accidents, yet findings regarding ISS and mortality remain inconsistent [10–12]. Orthopedic evaluations indicate that fracture distribution and extremity involvement may vary between electric and non-electric bicycle users [13, 14]. Collectively, these studies suggest that e-bike trauma may be characterized less by uniformly increased severity and more by a redistribution of regional injuries [9, 10, 15].

Beyond age and crash mechanics, anthropometric characteristics such as Body Mass Index (BMI) may represent an important but underexplored modifier of injury patterns. The global prevalence of overweight and obesity continues to rise substantially, with major implications for acute care systems [16, 17]. Obesity is characterized by chronic low-grade systemic inflammation, adipose tissue dysregulation, and altered immune signaling mediated by cytokines and alarmins such as High-Mobility Group Box 1 (HMGB1) [18]. These metabolic and inflammatory alterations may influence both the physiological response to trauma and the development of secondary complications [19, 20].

In trauma populations, obesity has repeatedly been associated with increased morbidity, prolonged hospitalization, and elevated risk of thromboembolic events, particularly among severely injured and geriatric patients [16–18]. A recent systematic review and meta-analysis demonstrated that severe obesity is associated with increased mortality and longer intensive care unit (ICU) stay following trauma [17]. Furthermore, obesity has been shown to modify orthopedic injury and fracture patterns in high-energy mechanisms such as motor vehicle collisions, suggesting altered biomechanical load transmission during impact [21–23]. These findings support the hypothesis that increased body mass may influence regional injury distribution independent of overall trauma severity.

Conversely, the concept of the “obesity paradox” has emerged from observational trauma research, proposing that overweight or moderately obese patients may demonstrate comparable or even improved survival after adjustment for confounders such as age and ISS [20, 24, 25]. Analyses of isolated blunt abdominal trauma and critical illness cohorts have suggested complex, non-linear associations between BMI and mortality [25]. Proposed mechanisms include altered metabolic reserves and modified inflammatory response, though biomechanical cushioning effects remain controversial [26, 27]. Nevertheless, while mortality and complication rates have been extensively studied, considerably less attention has been directed toward the influence of BMI on regional injury distribution in lower-energy mechanisms such as cycling accidents.

In cycling trauma, increased body mass may alter the center of gravity, fall dynamics, and energy transfer to the pelvis and extremities, potentially leading to mechanism-driven shifts in injury localization rather than increases in global ISS. Importantly, validated trauma scoring systems primarily quantify overall injury burden rather than mechanism-specific regional patterns [4], underscoring the need for detailed analysis of Abbreviated Injury Scale (AIS) distributions in relation to anthropometric factors.

Therefore, the present retrospective single-center cohort study aimed to compare adult trauma patients presenting after e-bike and conventional bicycle accidents regarding BMI, overall injury severity, regional AIS patterns, and early treatment intensity. We hypothesize that e-bike riders represent a demographically distinct subgroup characterized by a significantly higher BMI and a shifted pattern of pelvic and extremity injuries, without a corresponding increase in overall trauma severity or acute healthcare utilization. By integrating demographic characteristics, validated severity metrics, and multivariate regression analyses, this study seeks to clarify whether observed differences in injury patterns are primarily driven by rider-specific anthropometric factors rather than bicycle type alone.

## Materials and methods

The study was conducted in accordance with the Declaration of Helsinki (as revised in 2013) [28]. The protocol was reviewed and approved by the authors` local ethics committee and Institutional Review Board (approval No. **24–1512-S1-retro**). Given the retrospective nature of this analysis of existing patient records, the requirement for individual patient informed consent was waived by the Ethics Committee. All patient data were anonymized and de-identified prior to analysis. This study followed the STROBE guidelines for observational studies (Strengthening the Reporting of Observational Studies in Epidemiology) and the RECORD guidelines (Reporting of studies Conducted using Observational Routinely collected Data) [29, 30].

A retrospective single-center cohort study at our Level I trauma center was conducted. The study included all consecutive adult patients admitted to the trauma bay following bicycle-related accidents between 01/2018 and 12/2024. Patients were identified via a retrospective query of the hospital’s electronic medical records and trauma registry, in which trauma patients have been prospectively registered with their respective ISS. Inclusion criteria were: (1) age ≥ 18 years; (2) admission via the trauma bay following a bicycle-related accident (conventional bicycle or e-bike); and (3) completion of whole-body CT as part of the standardized trauma bay protocol. There was no ISS-based exclusion criterion; patients were included regardless of final ISS score. Patients with incomplete injury documentation or missing bicycle type information were excluded. Further exclusion criteria comprised: (1) pregnancy, (2) active malignant disease, (3) life-shortening neurodegenerative disease, (4) incomplete documentation precluding ISS calculation or bicycle type identification, and (5) transfer from external hospitals with incomplete initial assessment data.

Trauma bay activation was triggered by predefined clinical criteria including hemodynamic instability, altered consciousness (GCS ≤ 13), high-energy mechanism of injury, or suspected severe injury requiring immediate multidisciplinary assessment. ISS is calculated retrospectively and was not used as an activation or inclusion criterion.

Patient demographic data including age, sex, height, weight, and BMI, were documented. Clinical characteristics including American Society of Anesthesiologists Physical Status Classification (ASA Score), mechanism of injury details (e-bike vs. conventional bicycle), and prehospital vital signs were recorded. Injury severity was quantified using the Injury Severity Score (ISS) and region-specific maximum Abbreviated Injury Scale (AIS) scores for head/neck, face, thorax/thoracic spine, abdomen/lumbar spine, pelvis, extremities, and external injuries. Admission laboratory parameters were extracted from electronic records at trauma bay presentation, including pH, base excess, lactate, hemoglobin, International Normalized Ratio (INR), Quick, and Partial Thromboplastin Time (PTT). Physiological burden was assessed by Simplified Acute Physiology Score II (SAPS II) and treatment intensity by Therapeutic Intervention Scoring System-10 (TISS-10). Resource utilization was quantified by length of hospital stay (LOS), ICU days, and ventilation days.

Specific injury patterns were documented in detail, including presence and location of fractures (skull, facial bones, clavicle, ribs, vertebrae, pelvis, extremities), intracranial hemorrhages (epidural, subdural, subarachnoid, intracerebral), and solid organ injuries. Laboratory parameters at admission were extracted from electronic records. All data were abstracted and transferred to an electronic spreadsheet for analysis.

E-bike was defined as a bicycle equipped with an electric motor providing pedal assistance, typically limited to 25 km/h (European Union regulations). Conventional bicycle was defined as a pedal-powered bicycle without electric motor assistance. Primary endpoints were the overall injury severity (ISS) and regional maximum AIS scores. Secondary endpoints were the physiological burden (SAPS II), resource utilization (hospital LOS, TISS-10), and specific injury patterns (intracranial hemorrhage, extremity fractures). For primary analysis, patients were stratified into two groups based on bicycle type:

1. Conventional Bicycle Group (Bike): Riders of non-motorized bicycles (*n* = 224).
2. E-Bike Group (E-Bike): Riders of electrically assisted bicycles (*n* = 37).

### Statistical analysis

All variables were assessed for normality using histograms and quantile–quantile (Q–Q) plots and formally tested using the Shapiro–Wilk test. Given the non-normal distribution of most continuous variables, descriptive statistics were summarized as medians with interquartile ranges (IQR) for continuous variables and as counts with percentages for categorical variables. Group comparisons of continuous variables were performed using the Mann-Whitney U test. Categorical variables were compared using Fisher’s exact test or Chi-square test as appropriate. Multivariate logistic regression was employed to identify independent predictors of specific injury outcomes. Three multivariate binary logistic regression models were therefore constructed: (1) intracranial hemorrhage (ICH), (2) extremity fractures, and (3) e-bike riding. For the ICH model, ISS was replaced by non-head injury severity (maximum AIS score excluding the head region) to avoid circularity, as ISS incorporates AIS head scores. Covariates for each model were selected based on clinical relevance and univariate associations (*p* < 0.2). Event counts and EPV values are reported for each model in the Results section. Results are reported as odds ratios (OR) with 95% confidence intervals (95% CI). Effect sizes were calculated using Cohen’s d for continuous variables and Cramér’s V for categorical variables. Cohen’s d was interpreted as small (0.2–0.49), medium (0.5–0.79), and large (≥ 0.8) effects, while Cramér’s V was interpreted as small (0.1–0.29), medium (0.3–0.49), and large (≥ 0.5) associations. Post hoc power analysis was performed for the primary outcome (BMI difference between groups) using normal approximation methods for non-parametric tests. Power calculations were performed using custom Python scripts based on normal distribution approximations. All statistical tests were two-tailed with a significance level of *p* < 0.05. Confidence Intervals were reported where appropriate. Post-hoc power analysis or the ISS comparison of 17.4% indicates that the study was underpowered to detect moderate between-group differences in ISS. The absence of a statistically significant ISS difference should therefore be interpreted as “no evidence of a difference” rather than evidence of equivalence. Given the e-bike group size of *n* = 37, all regression models are considered exploratory. All statistical analyses were performed using GraphPad Prism version 10.6 (GraphPad Software, San Diego, CA, USA) and SPSS Statistics version 28.0 (IBM Corporation, Armonk, NY, USA).

## Results

### Patient demographics and baseline characteristics

**Fig. 1:**
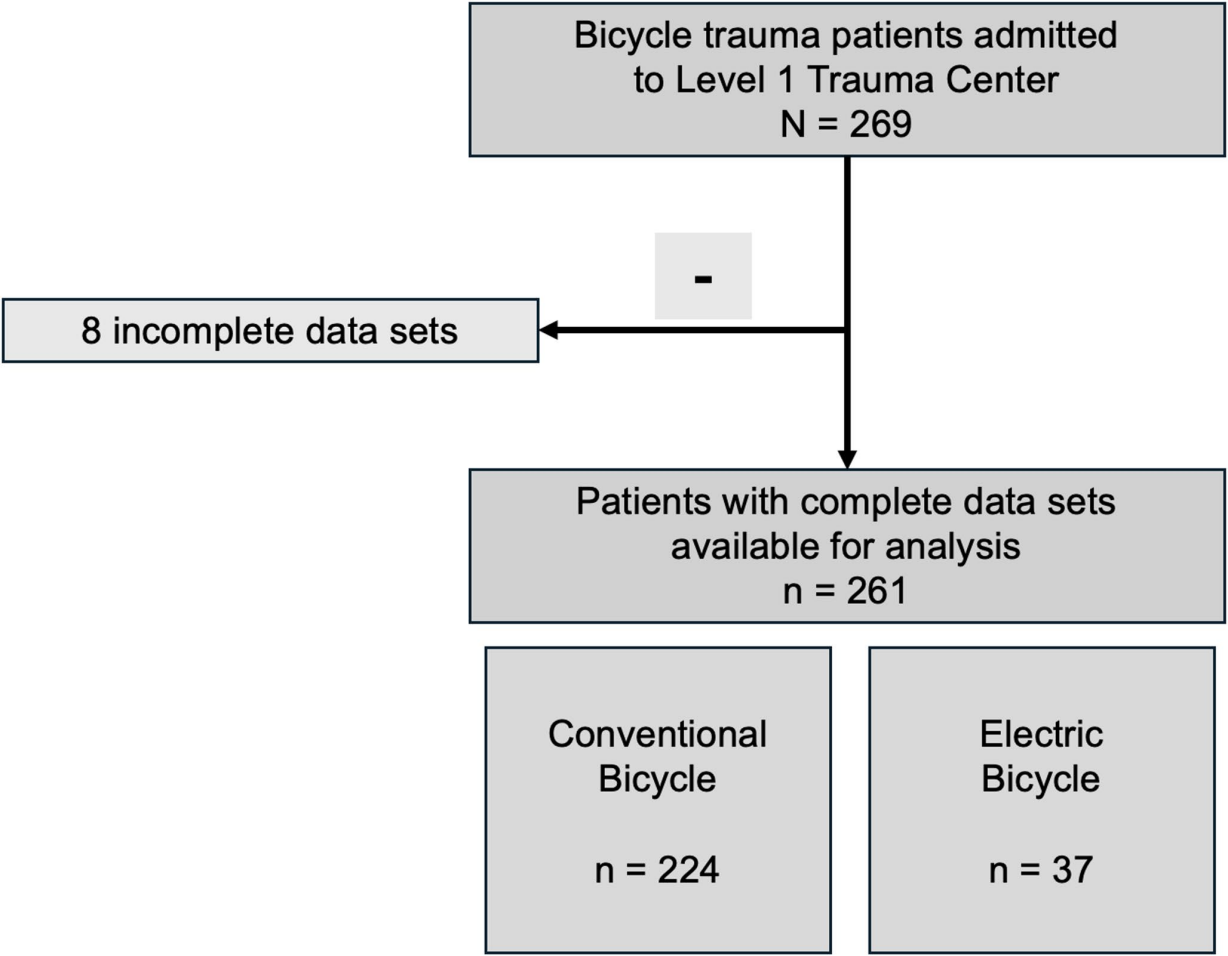
Patient flow chart illustrating flow for inclusion in our final data set

A total of 261 adult patients meeting the inclusion criteria were analyzed, including 224 conventional bicycle riders (85.8%) and 37 e-bike riders (14.2%) (Fig. 1).

**Fig. 2:**
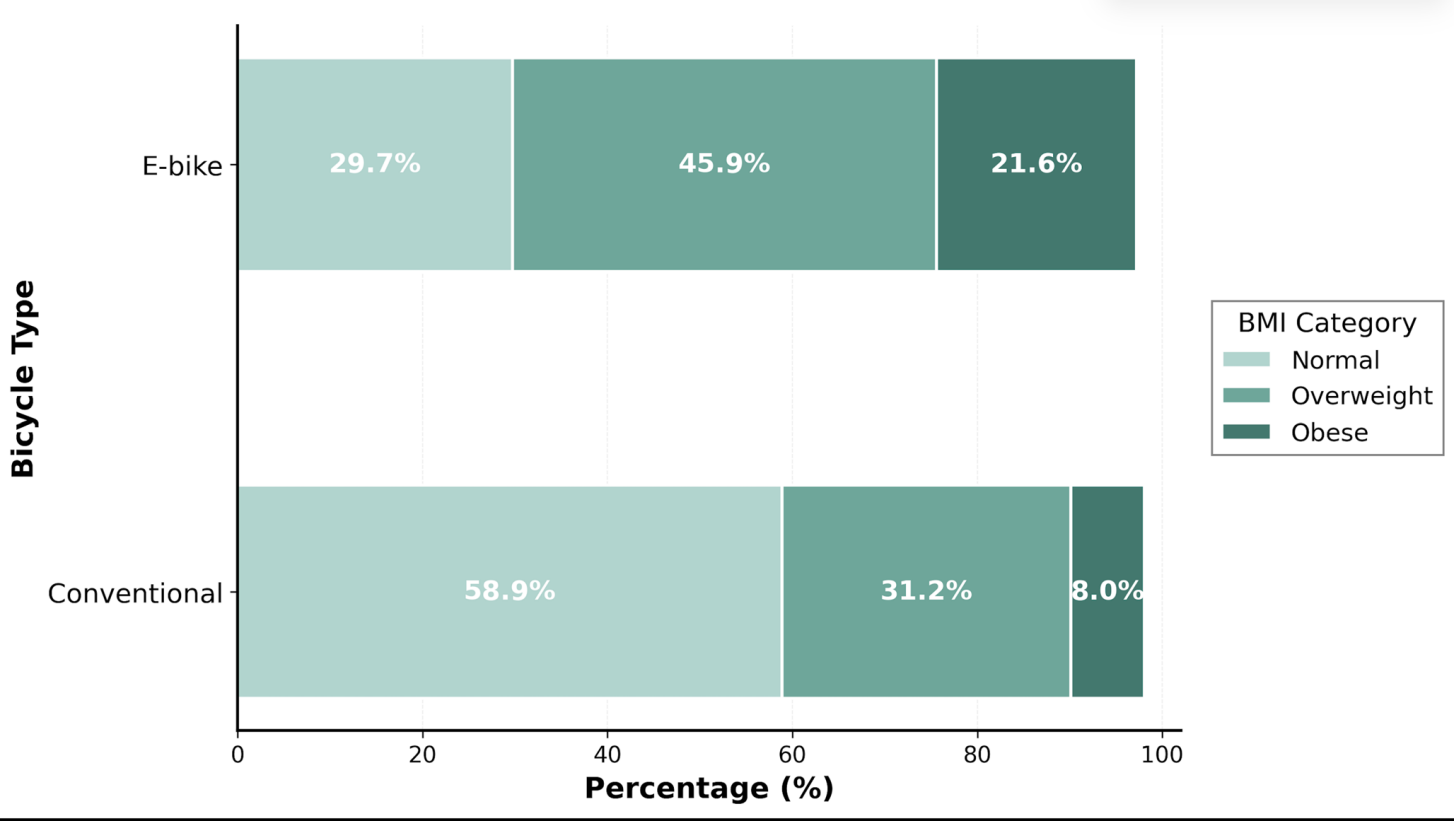
Bar chart, demonstrating BMI category distribution by bicycle type, differentiating between normal weight (light green bars, BMI < 25 kg/m²), overweight (green bars, BMI ≥ 25 kg/m²), and obese (dark green bars, BMI ≥ 30 kg/m²) in conventional and E-bike cyclists

Detailed demographic and clinical characteristics are presented in Table 1. E-bike riders had a significantly higher BMI (26.1 kg/m² vs. 24.6 kg/m²; *p* = 0.029). However, the absolute difference of approximately 1.5 kg/m² is modest. Both group medians fall within the normal-to-overweight range, and this difference is best interpreted as reflecting demographic self-selection rather than a clinical difference in injury risk. The ISS ≥ 16 rate was comparable between groups (46.9% vs. 43.2%; *p* = 0.680), and no statistically significant differences were observed in age, sex, ASA score, ICU-LOS, or hospital LOS. BMI category distribution differed significantly between groups (χ² = 13.56, df = 3, *p* = 0.004, Cramer’s V = 0.23; Fig. 2). Among conventional cyclists, 59% were normal weight (BMI < 25 kg/m²), 31% overweight (BMI ≥ 25 kg/m²), and 8% obese (BMI ≥ 30 kg/m²). In contrast, among e-bike riders, only 30% were normal weight, while 46% were overweight and 22% obese. This represents a substantial demographic shift, with e-bike riders approximately 2.7 times more likely to be obese than conventional cyclists.

Although e-bike riders tended to be older (median 59 years vs. 53 years), this difference did not reach statistical significance (*p* = 0.128) and showed only a small effect size (Cohen’s d = -0.21). ASA scores, reflecting baseline comorbidity burden, were comparable between groups (median 2.0 in both groups; *p* = 0.110).

Table 1 presents patient characteristics with median [IQR] for all continuous variables, integer reporting for ordinal scales (ASA), and additional variables requested by reviewers (sex distribution, ISS ≥ 16 counts, ICU-).

**Table 1:** Patient characteristics

Variable		Conventional bicycle (*n* = 224)		E-bike (*n* = 37)		*p*-value
Age (years), median [IQR]		53 [35–65]		59 [43–68]		0.128
Sex (male), n (%)		172 (76.8%)		25 (67.6%)		0.247
BMI (kg/m²), median [IQR]		24.6 [22.6–26.8]		26.1 [23.2–29.4]		0.029*
ASA score, median [IQR]†		2 [1–2]		2 [2–3]		0.116
ISS, median [IQR]		14 [6–24]		13 [6–22]		0.428
ISS ≥ 16, n (%)		105 (46.9%)		16 (43.2%)		0.680
ICU-admission, n (%)		138 (61.6%)		24 (64.9%)		0.855
ICU-LOS (days), median [IQR]‡		5 [3–12]		6 [3–13]		0.714
Hospital LOS (days), median [IQR]		11 [6–16]		10.5 [5–17]		0.618

*Differences between groups were statistically significant. †Median reported as integer. ‡ICU-LOS reported for patients admitted to ICU only

### Overall injury severity and physiological burden

Median ISS was similar between e-bike and conventional bicycle groups (13.0 vs. 14.0 points; *p* = 0.428), indicating no significant difference in overall injury severity. The effect size was negligible (Cohen’s d = 0.18), confirming that any difference in injury severity is clinically meaningless. Both groups demonstrated comparable proportions of patients with ISS ≥ 16 (major trauma). SAPS II scores, reflecting acute physiological derangement, showed no significant difference between groups (median 34.5 vs.5.0 points; *p* = 0.403, Cohen’s d = -0.12).

### Resource utilization

Total hospital LOS was comparable between e-bike and conventional bicycle groups (median 10.5 vs. 11.0 days; *p* = 0.452). Early treatment intensity, quantified by TISS-10 scores during the first 72 h, did not differ significantly between groups (median 15.0 vs. 14.5 points; *p* = 0.852, Cohen’s d = 0.02, negligible effect).

### Regional injury patterns (AIS scores)

Detailed regional AIS comparisons are presented in Table 2. Although median AIS Pelvis/Extremities scores were identical between groups (both median 2 [IQR 1–2]), the statistically significant Mann-Whitney U result (*p* = 0.045) reflects differences in the overall rank distribution rather than central tendency alone. Conventional cyclists had a higher proportion of patients with AIS Pelvis/Extremities ≥ 3 (32/224, 14.3%) compared to e-bike riders (2/37, 5.4%), indicating a greater frequency of severe pelvic injuries in the conventional cycling group. No other regional AIS domain showed a statistically significant difference.

Table 2 presents regional AIS scores as integers with IQRs and includes AIS ≥ 3 counts per region for both groups.

**Table 2:** Regional AIS scores and injury severity

AIS region	Conv. bicycle (*n* = 224)	E-bike (*n* = 37)	*p*-value
AIS Head/Neck, median [IQR]†	2 [1–3]	2 [1–4]	0.612
AIS Head/Neck ≥ 3, n (%)	71 (31.7%)	14 (37.8%)	0.466
AIS Face, median [IQR]†	1 [1–2]	2 [1–2]	0.341
AIS Face ≥ 3, n (%)	21 (9.4%)	3 (8.1%)	0.999
AIS Thorax, median [IQR]†	3 [2–4]	2 [2–3]	0.198
AIS Thorax ≥ 3, n (%)	83 (37.1%)	8 (21.6%)	0.072
AIS Abdomen, median [IQR]†	2 [1–2]	2 [2–3]	0.487
AIS Abdomen ≥ 3, n (%)	11 (4.9%)	2 (5.4%)	0.999
**AIS pelvis/Extremities**,** median [IQR]†**	**2 [1–2]**	**2 [1–2]**	**0.045**
AIS Pelvis/Extremities ≥ 3, n (%)	32 (14.3%)	2 (5.4%)	0.131
SAPS II, median [IQR]	33 [24–37]	35 [33–40]	0.403
TISS-10, median [IQR]	15 [10–15]	15 [10–19]	0.521

^*^Differences between groups were statistically significant. † Medians reported as integers

### Admission laboratory parameters

All admission laboratory parameters were comparable between groups without statistically significant differences, confirming similar physiological status at trauma bay presentation (Table 3).

Table 3 presents laboratory parameters raised in the trauma bay compared between both groups. Differences between groups were not significant (*p* > 0.05 for all).

**Table 3:** Laboratory parameters

Parameter	Conventional bicycle	E-bike	*p*-value
pH, median [IQR]	7.34 [7.30–7.38]	7.36 [7.31–7.39]	0.281
Lactate (mmol/L), median [IQR]	1.8 [1.4–2.4]	1.6 [1.1–2.3]	0.179
Haemoglobin (g/dL), median [IQR]	14.1 [12.9–15.1]	14.5 [12.9–15.7]	0.365
Base Excess (mmol/L), median [IQR]	-1.3 [-3.5-0.7]	-1.2 [-3.9-0.9]	1.000
INR, median [IQR]	1.04 [1.01–1.09]	1.03 [1.00-1.07]	0.177
Quick (%), median [IQR]	92 [82–100]	95 [86–105]	0.209
PTT (s), median [IQR]	25 [23–28]	26 [25–29]	0.124

### Age-stratified analysis

**Fig. 3:**
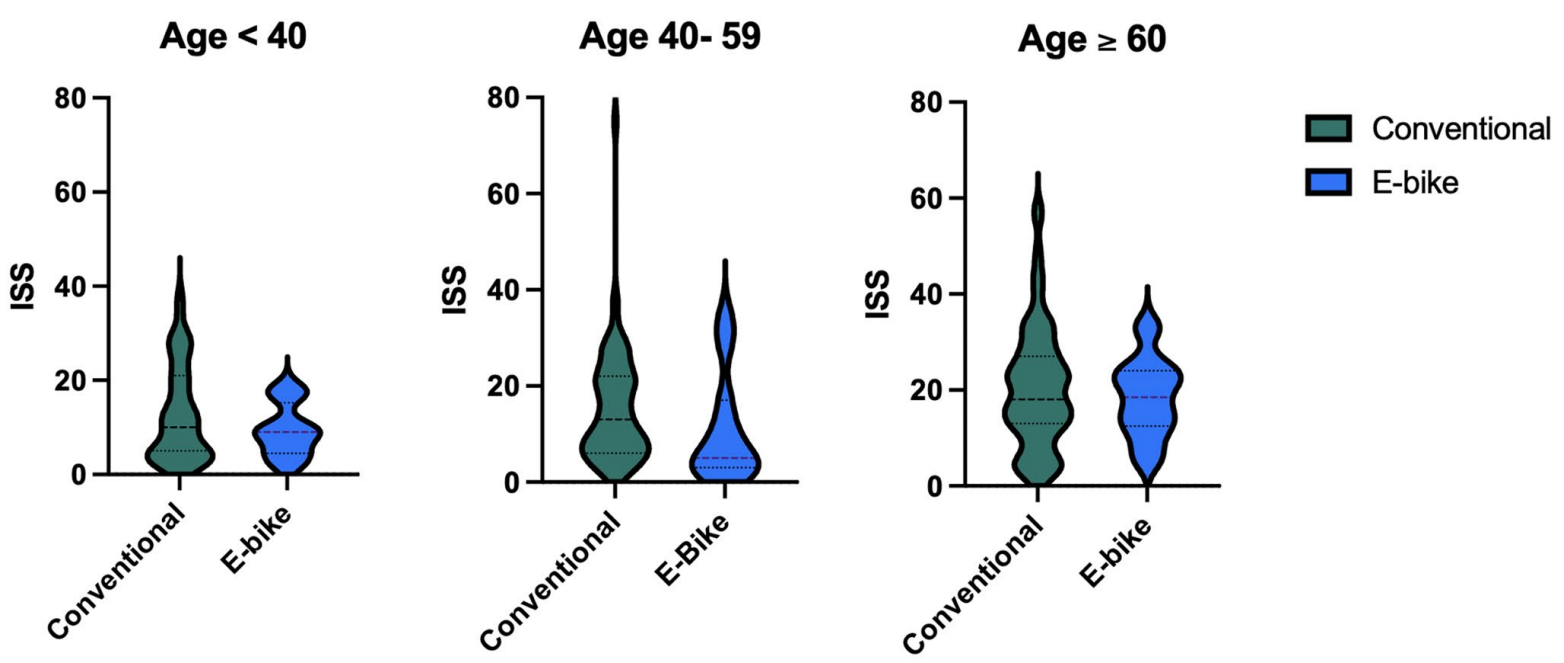
Violin-plot demonstrating age stratified distribution of injury severity score (ISS) by bicycle type, comparing conventional bike (Green painted violins) and E-bike (Blue painted violins), represented in medians (dashed lines) and their interquartile ranges (dotted lines)

Age-stratified analysis (Fig. 3) revealed that among younger riders (< 40 years), e-bike riders had notably higher BMI (27.6 vs. 24.0 kg/m²), yet similar ISS (median 9.0 vs. 10.0) compared to conventional cyclists. Among middle-aged riders (40–59 years), e-bike riders showed lower ISS (median 5.0 vs. 13.0), despite higher BMI. Among older riders (≥ 60 years), both BMI and ISS were similar between groups, suggesting that age emerges as the main factor in this population.

### Specific injury patterns in E-bike cohort

**Fig. 4:**
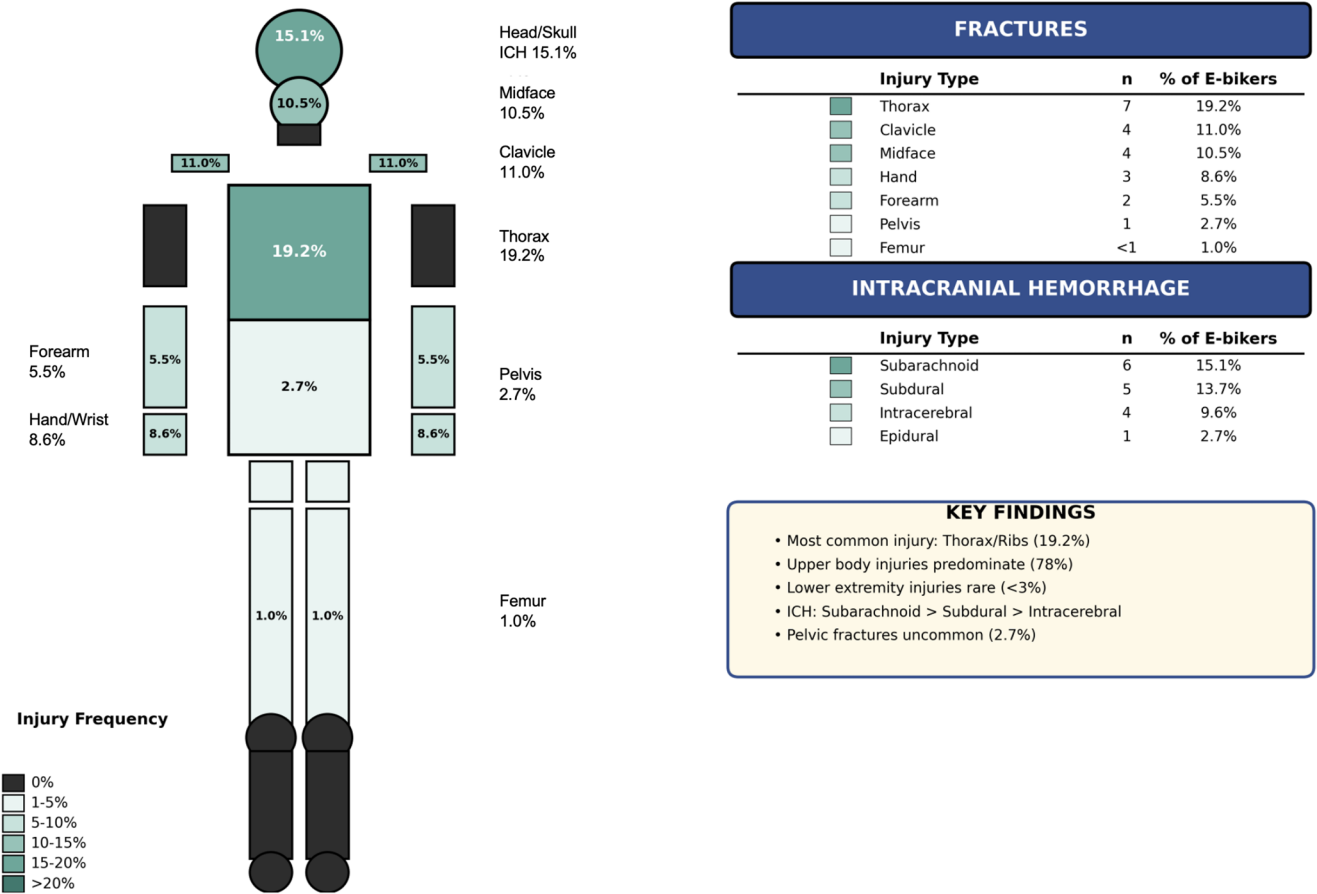
Injury distribution heatmap for e-bike riders. Cell shading reflects the relative frequency of each injury type, with lighter shades indicating lower frequency and darker shades indicating higher frequency. Exact proportions (%) are labeled within each cell. Anatomical regions are ordered craniocaudally (head/skull above midface)

The most common specific injuries observed in the e-bike cohort (*n* = 37) are illustrated in Fig. 4. The most frequent fractures were thoracic (19.2%), clavicular (11.0%), and midfacial (10.5%). Hand fractures occurred in 8.6% and forearm fractures in 5.5% of e-bike riders. Notably, pelvic fractures were relatively uncommon (2.7%), as were femoral fractures (1.0%). Subarachnoid hemorrhage was the most common intracranial bleeding pattern (15.1%), followed by subdural hematoma (13.7%) and intracerebral hemorrhage (9.6%). Epidural hematomas were rare (2.7%).

### Multivariate predictors of specific injury patterns

In the extremity fracture model, ISS (OR 1.042, 95% CI 1.014–1.071; *p* = 0.003) and BMI (OR 0.901, 95% CI 0.840–0.967; *p* = 0.004) were independent predictors. Each unit increase in ISS was associated with a 4.2% increase in odds of extremity fracture, while each 1 kg/m² increase in BMI was associated with a 9.9% reduction in odds — consistent with a modest protective effect of adiposity on extremity fracture risk. Age was not an independent predictor (OR 1.012; *p* = 0.134) (Table 4).

Table 4 presents three multivariate binary logistic regression models to identify independent predictors of specific injury outcomes: 1) intracranial hemorrhage (ICH), (2) extremity fractures, and (3) e-bike riding. To avoid circularity, ISS was replaced by non-head injury severity (maximum AIS score excluding the head region) in the ICH model. Regional maximum Abbreviated Injury Scale (AIS), Injury Severity Score (ISS) and Body Mass Index (BMI).

**Table 4:** Multivariate logistic regression

Dependent variable (model type)	Independent variable	Odds ratio (OR)	95% Confidence interval (CI)	*p*-value
Extremity fractures	Patient age	1.012	0,996–1.027	0.134
	**ISS**	**1.042**	**1**,**014–1.071**	0.003*
	**BMI**	**0**,**901**	**0.840–0.967**	0.004*
E-Bike riding	Patient age	1.015	0.994–1.037	0,165
	ISS	0.973	0.938–1.009	0.140
	**BMI**	**1.130**	**1.037–1.23**	0.005*
Intracranial hemorrhage †	**Patient age**	**1.033**	**1.017–1.050**	< 0.001*
	Non-head AIS	1.041	0.806–1.344	0.759
	BMI	0.984	0.915–1.059	0.674

*Differences between groups were statistically significant. † ICH model uses non-head AIS (max AlS excluding head) instead of ISS to avoid

#### Event counts and EPV values per model

ICH model (*n* = 261): ICH events = 80 (30.7%); 3 predictors; EPV ≈ 26.7 — adequate. Extremity fracture model (*n* = 261): events = 168 (64.4%); 3 predictors; EPV ≈ 56 — adequate. E-bike riding model (*n* = 261): e-bike events = 37; 3 predictors; EPV ≈ 12.3 — marginally adequate; interpret cautiously.

All models are considered exploratory given the overall sample size.

BMI was the sole independent predictor of e-bike riding (OR 1.130 per kg/m², 95% CI 1.037–1.230; *p* = 0.005), indicating that each 1 kg/m² increase in BMI was associated with a 13% increase in odds of riding an e-bike rather than a conventional bicycle. Age (OR 1.015; *p* = 0.165) and ISS (OR 0.973; *p* = 0.140) were not independent predictors.

In the ICH model, age was the only independent predictor (OR 1.033 per year; 95% CI 1.017–1.050; *p* < 0.001). Non-head AIS (OR 1.041; *p* = 0.759) and BMI (OR 0.984; *p* = 0.674) were not independently associated with ICH.

## Discussion

This retrospective single-center cohort study of 261 adult trauma patients compared injury patterns between e-bike and conventional bicycle accidents. Our principal findings challenge several common assumptions regarding e-bike trauma: (1) e-bike riders demonstrated significantly higher BMI compared to conventional cyclists, yet (2) overall injury severity (ISS), physiological burden (SAPS II), and resource utilization (LOS, TISS-10) were comparable between groups, (3) conventional cyclists exhibited higher pelvic injury severity, with no other regional AIS differences, and (4) multivariate analysis identified age as the sole independent predictor of intracranial hemorrhage; ISS and BMI independently predicted extremity fractures — with BMI showing a protective (inverse) association — while bicycle type predicted neither outcome. These findings suggest that injury patterns in cycling trauma are driven primarily by overall trauma severity and patient age rather than bicycle type or anthropometric characteristics.

### E-bike riders: A distinct demographic profile

E-bike riders in this cohort had a significantly higher BMI than conventional cyclists (26.1 vs. 24.6 kg/m²; *p* = 0.029). However, the absolute difference of approximately 1.5 kg/m² is modest and of uncertain clinical relevance. Both group medians fall within the normal-to-overweight range, and this difference alone is unlikely to have direct implications for acute trauma management. The finding is best interpreted as reflecting demographic self-selection - individuals with higher body weight or physical limitations preferentially adopting e-bikes to maintain cycling mobility [7–10, 31, 32] - rather than as a clinically actionable difference in injury risk. BMI was the sole independent predictor of e-bike riding (OR 1.13 per kg/m²; *p* = 0.005), though this result should be interpreted cautiously given the marginal EPV (≈ 9) for this model.

Although the observed BMI difference is statistically significant, the absolute magnitude (approximately 1.5 kg/m²) represents a shift from upper normal weight to lower overweight categories. This modest difference may explain why BMI did not emerge as an independent predictor of injury patterns in our multivariate analysis. Previous research in high-energy trauma mechanisms, such as motor vehicle collisions, has demonstrated that obesity can modify fracture patterns through altered biomechanical load transmission [33, 34]. However, our findings suggest that in lower-energy mechanisms like cycling accidents, the influence of BMI on injury distribution may be less pronounced or overshadowed by other factors such as overall trauma severity and age.

The trend toward older age in e-bike riders (median 59 vs. 53 years, *p* = 0.128), although not statistically significant in our cohort, aligns with previous epidemiological studies [31]. This age difference likely reflects both the physical assistance provided by electric motors and the growing adoption of e-bikes among older adults seeking to maintain mobility and independence. The combination of higher BMI and advanced age in e-bike users creates a distinct risk profile that warrants consideration in prevention strategies and clinical assessment.

### Comparable overall injury severity despite perceived risk

Contrary to widespread clinical perception and some previous reports [2], we observed no significant difference in overall injury severity between e-bike and conventional bicycle trauma. Median ISS values were nearly identical (13.0 vs. 14.0 points; *p* = 0.428). Physiological burden quantified by SAPS II was comparable (34.5 vs. 33.0 points; *p* = 0.403) as were all measures of resource utilization. The consistency of negligible effect sizes across all severity and resource utilization metrics strengthens our conclusion that e-bike trauma does not impose greater acute care burden than conventional cycling accidents. These findings are consistent with recent large-scale analyses suggesting that while e-bike crashes may result in higher hospitalization rates, global severity scores do not consistently demonstrate increased injury severity [7, 9, 10].

Several factors may contribute to the comparable injury severity observed in our study. First, e-bike speeds in urban and recreational settings, while higher than conventional bicycles, typically remain substantially lower than motorcycles or motor vehicles, limiting the kinetic energy involved in crashes [7, 9, 35]. Second, many jurisdictions have implemented speed limitations for e-bikes (25 km/h in the European Union, 32 km/h for faster Swiss e-bikes), which may mitigate severity [7]. Third, the older age and potentially more cautious riding behavior of e-bike users may offset any speed-related risk increase [9, 10].

The comparable resource utilization between groups (LOS and TISS-10) further supports the conclusion that e-bike trauma does not impose a greater acute care burden than conventional cycling accidents. This finding has important implications for trauma system planning and resource allocation, suggesting that the rapid expansion of e-bike usage should not necessitate disproportionate increases in trauma care capacity.

However, this finding must not be interpreted as evidence of equivalence. The post-hoc power for the ISS comparison was only 17.4%, meaning the study was underpowered to detect moderate between-group differences. The appropriate interpretation is that there was “no evidence of a difference” given the available sample size, not that no difference exists. All conclusions regarding injury severity should be regarded as hypothesis-generating and require confirmation in larger prospective studies.

### Shifted rather than intensified injury patterns

Our regional AIS analysis revealed a nuanced pattern: conventional cyclists demonstrated significantly higher pelvic injury severity (*p* = 0.045), while no other body regions showed significant between-group differences. This finding contradicts the hypothesis that e-bike riders would exhibit more severe injuries due to higher impact velocities. Instead, it suggests that injury patterns are shifted rather than uniformly intensified.

The higher pelvic injury severity in conventional cyclists (*p* = 0.045) may reflect distinct differences in biomechanical loading and cycling posture. Conventional bicycles, particularly those used for commuting or competitive athletic cycling, often require a more aerodynamic, “hunched” posture characterized by increased trunk flexion and anterior pelvic tilt in order to reach lower handlebar positions and optimize aerodynamic efficiency [36]. This riding position may alter the orientation of the pelvis relative to the ground and could place the acetabulum in a more direct alignment with the direction of impact during a side-impact or lateral fall. In cycling trauma, acetabular fractures have been reported following direct lateral impact onto the hip, with force transmission through the greater trochanter to the acetabular dome representing a plausible injury mechanism [37].

Conversely, many e-bike designs—often described as “city” or “commuter” frames—encourage a more upright riding posture with reduced trunk flexion and a more neutral spinal and pelvic alignment. This more upright positioning may influence the rider’s center of gravity and modify the vectors of kinetic energy dissipation during a fall, which could effectively spare the pelvis from the high-energy axial loading often seen in conventional cycling accidents. Furthermore, epidemiological studies suggest that fracture patterns and injury severity may differ depending on the cycling context, with sports-oriented cycling being associated with a higher proportion of high-energy trauma compared with more utilitarian forms of cycling [38]. In addition, electric bicycle use has been associated with different rider demographics and a more utilitarian usage compared with conventional bicycles, factors that may further contribute to differences in accident mechanisms and injury profiles [9]. Taken together, these biomechanical and epidemiological considerations may provide a plausible explanatory framework for the differences observed in the present study and should therefore be interpreted as potential mechanisms rather than definitive causal relationships.

This mechanistic explanation requires prospective validation with crash reconstruction data to be further established.

### Independent predictors: Severity and age, not bicycle type

Our multivariate logistic regression analyses provide crucial insights into the determinants of specific injury patterns. For intracranial hemorrhage, patient age emerged as the sole independent predictor (OR 1.033 per year; *p* < 0.001), while non-head AIS and BMI were not independently associated. This is biologically plausible, as older patients have greater cerebrovascular fragility and reduced tolerance to head trauma [15].

ISS and BMI were independent predictors of extremity fractures (OR 1.042 and OR 0.901, respectively; both *p* < 0.01). The inverse association between BMI and extremity fractures — each 1 kg/m² increase in BMI associated with approximately a 10% reduction in odds — may reflect the cushioning effect of adipose tissue on extremity loading, consistent with observations in motor vehicle and pedestrian trauma [33, 34]. Age was not an independent predictor in this model (OR 1.012; *p* = 0.134), though this may reflect the limited power of the e-bike subgroup.

### The “E-bike risk” may be overestimated

Our findings collectively suggest that the perceived “e-bike risk” in clinical and public discourse may be overestimated or misdirected. While e-bike riders constitute a demographically distinct group (higher BMI, trend toward older age), they do not demonstrate increased overall injury severity, treatment burden, or consistently more severe regional injuries compared to conventional cyclists. The significant difference in pelvic injury severity favored e-bike riders (lower severity), contradicting expectations of uniformly worse outcomes.

This apparent paradox may be explained by several mechanisms. First, e-bike riders may exhibit more cautious behavior, potentially related to older age and awareness of their physical limitations [32]. Second, the electric assistance may enhance stability and control, particularly at lower speeds and during hill climbing, potentially reducing fall risk in certain scenarios. Third, the demographic shift toward older, higher-BMI users may be accompanied by different riding contexts (recreational paths vs. urban traffic) with distinct risk profiles [39].

From a public health perspective, these findings suggest that e-bikes should not be stigmatized as inherently dangerous compared to conventional cycling. The substantial health benefits of increased cycling participation, particularly among older adults and those with physical limitations, may outweigh modest injury risks. Policy interventions should focus on evidence-based safety measures (helmet use, dedicated cycling infrastructure, speed regulations) rather than discouraging e-bike adoption based on overstated injury concerns [15, 40].

While public discussion frequently focuses on e-bikes as a distinct safety concern, our findings did not identify clinically meaningful differences in overall injury severity, ICU utilization, or hospital length of stay between e-bike riders and conventional cyclists within this trauma-bay cohort. These observations suggest that once severely injured cyclists require trauma-bay evaluation, acute injury burden and resource utilization appear broadly comparable between rider groups.

### BMI and the obesity paradox in cycling trauma

The absence of BMI as an independent predictor of injury patterns in our multivariate analysis warrants discussion in the context of the broader obesity literature in trauma. While obesity has been consistently associated with increased morbidity and complications in high-energy trauma [17], the relationship may be more complex in lower-energy mechanisms like cycling accidents.

Our findings provide partial support for a protective BMI effect in cycling trauma: BMI was an independent inverse predictor of extremity fractures (OR 0.901; *p* = 0.004), consistent with an adipose cushioning mechanism. However, BMI was not associated with ICH or overall injury severity, suggesting the effect is anatomically specific rather than reflecting a general obesity paradox.

The modest BMI difference observed (1.5 kg/m²) may simply be insufficient to exert meaningful biomechanical or physiological effects on injury patterns. Alternatively, potential protective effects of adipose tissue cushioning [20] may be counterbalanced by altered fall dynamics and increased kinetic energy in heavier individuals.

The inflammatory and metabolic alterations associated with obesity [17, 19, 20] may primarily influence secondary complications (thromboembolism, wound healing, infections) rather than acute injury patterns. Our study design, focused on initial injury severity and early resource utilization, was not optimized to detect such delayed effects. Future prospective studies with longer follow-up periods would be valuable to comprehensively assess obesity’s impact across the entire trauma care continuum in cycling injuries.

### Clinical implications

Our findings have several practical implications for trauma care:

1. Risk stratification: Clinicians should avoid assuming greater injury severity based solely on e-bike involvement. Standard trauma assessment protocols, emphasizing ISS calculation and age-specific risk factors, remain appropriate.
2. Resource allocation: Trauma systems need not allocate disproportionate resources specifically for e-bike trauma, as acute care burden appears comparable to conventional cycling injuries.
3. Age-specific protocols: Given age as the sole independent predictor of ICH, heightened vigilance for intracranial injury in older cyclists is warranted. Regarding extremity fractures, overall trauma severity (ISS) is the primary driver; a higher BMI was associated with reduced — not increased — extremity fracture risk.
4. Patient counseling: Healthcare providers can reassure patients that e-bike use does not inherently confer greater injury risk compared to conventional cycling, potentially encouraging active mobility among older adults and those with physical limitations.

### Strengths and limitations

This study’s strengths include the consecutive patient cohort, comprehensive injury documentation using validated severity scores (ISS, AIS), assessment of both overall and regional injury patterns, and multivariate analysis adjusting for potential confounders. The single-center design ensured uniform assessment and documentation protocols.

The primary limitation of this study is the relatively small sample size of the e-bike cohort (*n* = 37), which resulted in a post-hoc power of 17.4% for the comparison of global injury severity (ISS). Consequently, the lack of statistically significant differences in ISS and resource utilization between groups should be interpreted as an absence of evidence for a difference rather than proof of equivalence. All multivariate models are exploratory. The EPV for the e-bike riding model (≈ 9) falls marginally below the conventional threshold of 10, and all regression findings carry wide confidence intervals with uncertain stability. The ICH and extremity fracture models achieved adequate EPV for the full cohort (≈ 24 and well above 10, respectively). The e-bike riding model (EPV ≈ 9) is marginally underpowered. All models should be considered hypothesis-generating. The original model including ISS as a predictor of ICH was methodologically flawed due to the partial overlap between ISS and AIS head. The revised model addresses this limitation, but the corrected findings also carry the general caveats regarding sample size and exploratory status.

An important limitation relates to cohort selection. The present study included only cyclists who triggered trauma-bay activation and underwent whole-body CT imaging. Consequently, the cohort represents a highly selected subgroup of injured cyclists rather than the overall cycling population. Potential differences in trauma-bay activation thresholds, referral patterns, or prehospital triage between e-bike riders and conventional cyclists could introduce selection bias. Therefore, our findings should not be interpreted as estimates of the overall risk of severe injury associated with either bicycle type.

This study exclusively included patients admitted via the trauma bay and undergoing whole-body CT. Mild cycling injuries - which represent the majority of bicycle-related trauma in population-based studies - are systematically excluded. The findings apply strictly to the subset of cyclists sustaining injuries severe enough to trigger trauma bay activation and must not be extrapolated to the general cycling population or used to make statements about e-bike risk in the broader context of cycling safety.

The study lacked data on specific injuries of the conventional cycling cohort, helmet use, crash velocity, riding environment (urban vs. recreational), crash counterpart (single-vehicle fall vs. collision), and road surface conditions. These are known determinants of cycling injury severity, and their absence represents an irreducible limitation of the retrospective registry-based design. The observed lack of significant differences between groups may partly reflect unmeasured behavioral differences (e.g., differential helmet use) rather than inherent equivalence of injury risk. Prospective studies with standardized crash documentation are needed.

Furthermore the retrospective single-center design introduces selection and documentation biases. Generalizability may be limited to settings with similar e-bike regulations and riding cultures. Long-term outcomes were not assessed.

## Conclusions

This retrospective cohort study of 261 adult trauma bay patients found no statistically significant differences in overall injury severity, physiological burden, or acute care resource utilization between e-bike and conventional cycling trauma in this selected population. E-bike riders had a significantly higher BMI, which was the sole independent predictor of e-bike riding — consistent with demographic self-selection — though the absolute BMI difference is modest and of uncertain clinical relevance. ISS and BMI (inversely) independently predicted extremity fractures; age was the sole independent predictor of ICH. Conventional cyclists had a higher proportion of severe pelvic injuries. These findings suggest that, within a severely injured trauma-bay population, injury burden and acute treatment requirements are comparable between e-bike riders and conventional cyclists. The perceived “e-bike risk” may be overestimated in clinical assumptions and public discourse. Rather than inherently dangerous, e-bikes appear to facilitate cycling participation among older adults and individuals with higher BMI, potentially expanding the health benefits of active transportation without disproportionate injury burden. Trauma care protocols should emphasize standard severity-based assessment and age-specific considerations rather than assuming greater risk based solely on e-bike involvement. These findings, however, are exploratory and hypothesis-generating. Future research should prioritize prospective multicenter cohort studies with long-term outcome assessment.

## Data Availability

No datasets were generated or analysed during the current study.
